# Designing a Clinician-Facing Tool for Using Insights From Patients’ Social Media Activity: Iterative Co-Design Approach

**DOI:** 10.2196/16969

**Published:** 2020-08-12

**Authors:** Dong Whi Yoo, Michael L Birnbaum, Anna R Van Meter, Asra F Ali, Elizabeth Arenare, Gregory D Abowd, Munmun De Choudhury

**Affiliations:** 1 School of Interactive Computing Georgia Institute of Technology Atlanta, GA United States; 2 The Zucker Hillside Hospital Northwell Health Glen Oaks, NY United States; 3 The Feinstein Institutes for Medical Research Manhasset, NY United States; 4 The Donald and Barbara Zucker School of Medicine at Hofstra/Northwell Hempstead, NY United States

**Keywords:** social media, psychotic disorders, information technology

## Abstract

**Background:**

Recent research has emphasized the need for accessing information about patients to augment mental health patients’ verbal reports in clinical settings. Although it has not been introduced in clinical settings, computational linguistic analysis on social media has proved it can infer mental health attributes, implying a potential use as collateral information at the point of care. To realize this potential and make social media insights actionable to clinical decision making, the gaps between computational linguistic analysis on social media and the current work practices of mental health clinicians must be bridged.

**Objective:**

This study aimed to identify information derived from patients’ social media data that can benefit clinicians and to develop a set of design implications, via a series of low-fidelity (lo-fi) prototypes, on how to deliver the information at the point of care.

**Methods:**

A team of clinical researchers and human-computer interaction (HCI) researchers conducted a long-term co-design activity for over 6 months. The needs-affordances analysis framework was used to refine the clinicians’ potential needs, which can be supported by patients’ social media data. On the basis of those identified needs, the HCI researchers iteratively created 3 different lo-fi prototypes. The prototypes were shared with both groups of researchers via a videoconferencing software for discussion and feedback. During the remote meetings, potential clinical utility, potential use of the different prototypes in a treatment setting, and areas of improvement were discussed.

**Results:**

Our first prototype was a card-type interface that supported treatment goal tracking. Each card included attribute levels: depression, anxiety, social activities, alcohol, and drug use. This version confirmed what types of information are helpful but revealed the need for a glanceable dashboard that highlights the trends of these information. As a result, we then developed the second prototype, an interface that shows the clinical state and trend. We found that focusing more on the changes since the last visit without visual representation can be more compatible with clinicians’ work practices. In addition, the second phase of needs-affordances analysis identified 3 categories of information relevant to patients with schizophrenia: symptoms related to psychosis, symptoms related to mood and anxiety, and social functioning. Finally, we developed the third prototype, a clinical summary dashboard that showed changes from the last visit in plain texts and contrasting colors.

**Conclusions:**

This exploratory co-design research confirmed that mental health attributes inferred from patients’ social media data can be useful for clinicians, although it also revealed a gap between computational social media analyses and clinicians’ expectations and conceptualizations of patients’ mental health states. In summary, the iterative co-design process crystallized design directions for the future interface, including how we can organize and provide symptom-related information in a way that minimizes the clinicians’ workloads.

## Introduction

### Background

Psychotic disorders, such as schizophrenia and schizoaffective disorder, constitute a serious menace to personal health [1]. Symptoms characterizing these conditions emerge during critical years of adolescence; interfere with the establishment of healthy social, educational, and vocational foundations; and are associated with devastating individual and familial loss [2]. However, there are no known cures for these conditions [3]. Once developed, they often become chronic and must be managed [4]. Subjective patient experiences in the form of clinical interviews, questionnaires, patient self-reports, and family observations have played a central role in the management of these conditions for over a century [5]. These approaches require that behavioral, emotional, or cognitive symptoms are recalled from a patient’s memory, a method prone to retrospective recall bias [6,7]. Patients also frequently withhold information from their clinicians because of stigma, guilt or shame, failure to remember relevant events, or to meet impression management goals [8]. In fact, the psychopathology of serious mental illnesses may affect the very mental, emotional, and social judgments on which patients’ self-reported responses are based [9]. Therefore, there is a need to access more objective information about patients to augment patients’ verbal reports in clinical settings.

In recent years, patient-generated data, machine learning, and digital health technologies have been recognized to offer unprecedented opportunities to help consumers, clinicians, researchers, and other stakeholders measure, manage, and improve mental health [10-12]. In particular, digital records of people’s activities on social media, such as Facebook and Twitter, have been shown to enable the development of machine learning algorithms that can assess and even predict risk to a variety of mental health challenges [10], such as major depression [13], postpartum depression [14], posttraumatic stress [15,16], and schizophrenia [17]. Research has also demonstrated that a variety of symptomatic expressions and outcomes of clinical interest (eg, depression risk or suicidal ideation) may be gleaned from such data [18,19]. Although social media platforms may negatively affect users’ behaviors (eg, being confined to echo chambers [20], exposure to misinformation [21], and increased social comparison [22]), they constitute voluntarily shared and recorded data that include the feelings, opinions, and daily experiences of their users. Therefore, with social media, it is possible to unobtrusively and longitudinally gather insights about people’s social interactions, affect, and cognitive styles—signals characteristic of one’s mental health state and thus critical to management and treatment of mental illnesses but which may be challenging to assess via psychometric or self-reported means because of concerns of impression management and retrospective recall bias [10].

Although researchers have argued that social media analysis can be used to support real-world mental health care settings, interventions, and clinical diagnosis and assessment [23,24], actual opportunities concerning such technology in the clinical setting have not been explored. As a way to bridge this gap, through this research, our ultimate goal is to develop a clinician-facing tool that can provide mental health insights derived from patients’ social media at the point of care. This future tool will collect patients’ social media data under their informed consent and analyze the collected data to distill clinically meaningful insights. The analyzed data will be shown in a way that can support mental health clinicians’ work practices without adding intellectual burden or information overload.

### Objectives

To achieve the aforementioned design goal, there are many challenges we need to overcome: we must understand the current work practices of mental health clinicians; we need to explore clinicians’ potential needs, barriers, and concerns regarding the concept of this future interface; and, most importantly, we need to introduce the concept of delivering mental health insights from patients’ social media to clinicians—a practice that currently does not exist. Therefore, as a first step toward conceptualizing this future technology, drawing on approaches in the human-computer interaction (HCI) field, we aimed to create a series of low-fidelity (lo-fi) prototypes informed by the clinicians’ opinions and needs regarding these insights gleaned from social media. These include whether social media analysis can be useful in the context of their clinical practices, what kinds of insights from patients’ social media data may be useful, and how these insights can be presented so as to be seamlessly integrated into their workflows in the future.

We, as an interdisciplinary research team, co-designed the lo-fi prototypes. Co-design, which is widely used in the design research community, can be defined as a dialog between end users and designers [25]. We extended the concept of co-design as a long-term discussion between end users and designers. The 4 clinical researchers in this team were the potential end users of the prototype. At the same time, they were also domain experts who could represent end users. The rest of the HCI researchers served as designers and builders of future tools that can support clinicians.

Over the 6 months of co-design activities, we adopted the needs-affordances analysis framework to support the iterative process. Needs-affordances analysis is a method to refine concrete design directions based on both what the users want to have (needs) and what the technology/platform can provide (affordances) [26].

We first discuss the concept of co-design and needs-affordances analysis as the method of this project. Then, we present the 3 different lo-fi prototypes that are the results of our co-design activities. Finally, we discuss the lessons learned from this project and future plans for building clinician-facing mental health technologies that are powered by patients’ social media data.

## Methods

### Iterative Co-Design Process

Our research explores the building of technologies to support clinicians in mental health treatment, specifically focusing on schizophrenia, as a case example. Schizophrenia is a chronic and severe mental disorder characterized by abnormal social behaviors and distorted perceptions of reality [2]. Clinical observations indicate that it is among the most challenging mental illnesses to manage, primarily because of its high likelihood of relapse. Most patients experience multiple relapses during the course of the illness [27]. Management of the illness, therefore, involves preventing the occurrence of a relapse as well as symptomatic exacerbation [28]. Previous research has demonstrated that we can computationally extract behavioral and linguistic patterns from social media posts of patients with schizophrenia [17,29]. These approaches help us to predict mental health states and clinical outcomes, such as relapse episodes in schizophrenia [30], or to identify a clinically valid diagnosis of the illness [31]. Given the rich evidence that social media can be helpful for understanding patients with schizophrenia, we deemed focusing on the context of schizophrenia, which allows us to examine whether such information may be used at the point of care through a technological interface.

These empirical evidences are further supported by recent research that has revealed patients’ interest in and willingness to use their social media data to improve their treatment outcomes. Padrez et al [24] found that as many as 70.4% (1008/1432) of patients who reported using social media and were willing to participate in research also agreed to share these data and have them linked to their medical records for health research. Similarly, Rieger et al [32] reported that in a survey of 238 mental health patients, of those who had a social media account and were receiving therapy, 74.4% (99/133) indicated that they would be willing to share their social media posts with their therapists, if their therapists were concerned about how they were doing, including 53% (50/94) who were willing to share both public and private posts. Specific to psychotic disorders and most relevant to our context, based on semistructured interviews with 112 psychotic disorder patients, in a work by Birnbaum et al [33], 80.7% (88/109) of patients were open to sharing their social media data with clinical researchers to explore how it can potentially inform their treatment. Drawing on these findings, and because clinical researchers have advocated that it is important to first focus on issues related to the accuracy, interpretability, meaning, and actionability of these data such as social media [34], in this paper, we focus on co-design exercises with clinicians. Our rationale is further bolstered by Baier’s [35] observation, “Without judicious consideration, social media use by [clinicians] can lead to inadvertent self-disclosures to [patients] that risk damaging the therapeutic alliance, interfering with therapeutic processes, and placing both the [patient] and clinician at risk”.

As mentioned earlier, this paper focuses particularly on the process of building a series of lo-fi prototypes toward the aforementioned goal. In its basic form, a prototype is an expression of design intent. Prototyping allows designers to present their designs and see them in action. In the context of digital technologies such as our case, a prototype is a simulation of the final interaction between the user (the clinician) and the interface (communicating insights from patients’ social media activities). lo-fi prototyping is a quick and easy way to translate high-level design concepts into tangible and testable artifacts. The first and most important role of lo-fi prototypes is to check and test functionality rather than the visual appearance of the product.

To develop our lo-fi prototypes, an interdisciplinary research team collaborated for 6 months to envision future technologies through an iterative co-design process (Table 1 provides the details of the team members). The team members spoke for roughly an hour via video conference calling every 2 weeks because the team was geographically distributed. In earlier meetings, we discussed experts’ user needs and the potential affordances of our future technology. Experts included clinicians working at a large health care system in the northeast of the United States. After we (HCI researchers at a large public university in the southeast of the United States) developed design sketches based on those discussions, we exchanged our ideas and thoughts while reviewing our sketches via the video conferencing app. During the discussion, one of the researchers wrote the points discussed and shared the notes with other researchers. The written notes mainly informed our design decisions during the iterative process, and the decisions were reflected during the subsequent meetings. This research was approved by the institutional review boards of the relevant institutions.

**Table 1:** Details of the backgrounds of the team members.

Types of researchers	Number of researchers	Job role	Types of work	Research experience, years
HCI^a^ researchers	3	1 PhD student, 2 Computer Science professors	Research in social media analytics, health sensing, and design	5-32
Clinical researchers	4	1 psychiatrist, 1 clinical psychologist, and 2 clinical research coordinators	Mental health care delivery to patients and clinical and intervention research	6-10

^a^HCI: human-computer interaction.

More elaborately, we adopted an iterative strategy, in which we created a design sketch led by the HCI researcher on the team, obtained feedback on it from the clinician collaborators, and then adjusted it accordingly to create a modified or new sketch. Over time, the clinicians felt insights from social media could work as *collateral information* [36,37], which could complement existing ways of gathering information from patients and thereby overcome some of their limitations. Collateral information, which is information obtained from sources such as family members, primary providers, and friends, in mental health is important because it may supply objective, finer-grained data; can facilitate time-sensitive monitoring of symptoms; and can provide an ability to detect subtle but burgeoning changes in a patient’s condition, eventually improving clinical assessments.

Furthermore, we postulated Facebook as our target social media platform for discussions surrounding the co-design process. This is because Facebook is widely adopted among young adults [38], and previous research has established that it is the most widely used social media site among the target patient population [23]. Moreover, in contrast to other social media platforms, Facebook provides higher identity and social affordances and has a rich set of social interaction capabilities [39], which allows the possibility to glean a variety of potentially relevant mental health insights.

The abovementioned iterative process of our co-design activities was open ended because of the exploratory nature of our study; however, we used the following questions as starting points when we evaluated our sketches:

- Do the visualizations support the clinicians’ needs that are identified by a needs-affordances analysis?
- Are the visualizations easy to understand?
- Will the visualizations be compatible with clinicians’ work practices?
- What are the more/less important categories?
- What are the other categories that can be helpful for clinicians?
- What are the areas of improvement?

On the basis of the feedback from these questions, we decided the direction of the next iterations. The details of the 3 iterations are described in *Results* section.

### Needs-Affordances Analysis

Affordance refers to the features of an object in the physical environment that may cause interactions between the object and an agent [26]. In other words, affordances basically represent the opportunities for action that an object offers. Moreno and D’Angelo [39] proposed an affordances framework to design platform-sensitive social media–based health interventions. They also presented empirical evidence supporting why the consideration of platform affordances can support future health intervention designs. We follow this line of thought: we use this framework to choose our target social media platform, Facebook, and then, we consider the affordances of Facebook to create our lo-fi prototypes for eventual tools that can be used to augment clinical care and treatment settings.

Antonenko et al [26] used the concept of affordances to enable researchers to design/evaluate new technologies that can support user needs. They proposed a needs-affordances analysis as a framework developed within the field of education research to design and evaluate new education technologies. It helped them to define user needs, identify a potentially appropriate technology, and align technological affordances with the specific needs of target users. Technological affordances are the possibilities of interactions a new technology can offer to potential users, which can be similar to the affordances of interventions in Moreno and D’Angelo’s framework.

In this study, we apply a needs-affordances analysis to the process of creating lo-fi prototypes. It plays 2 vital roles in the process. First, it provides a systematic structure to the discussion between HCI researchers and clinical researchers. This highlights the need for clinical researchers to define user needs to crystallize a future interface while providing opportunities for HCI researchers to explain what kinds of needs can be supported by social media data analysis. Second, although this analytical framework does not provide a detailed guideline for design decisions, its ability to reveal the alignment of needs and affordances provides a hint of what the prototypes should be.

The combination of iterative co-design and needs-affordances analysis gave us 3 prototypes: (1) a card-type interface for treatment goal tracking, (2) a clinical state and trend visualization interface, and (3) a clinical summary dashboard.

## Results

### A Card-Type Interface for Treatment Goal Tracking

On the basis of consensus in the early discussions that social media can be a source of collateral information, our first lo-fi prototype generation process began by exploring the needs of our possible user group: mental health clinicians. Clinical researchers, grounded in clinical theory and practice, made a list of common treatment goals [40] they care about in the real-world clinical setting (Table 2). We compiled a list of the different kinds of information and computational insights that can help to assess whether a patient is close to, progressing toward, or digressing from their treatment goals. Next, we discussed the affordances of our chosen social media platform (Facebook) that can be relevant to meeting these treatment goals. For example, social and emotional affordances, which are related to the opportunities to communicate with others and express their emotions on social media, can be relevant to assess whether the patients are increasing or decreasing their social interactions and/or sharing emotional vulnerability with their friends. On the basis of the affordances of Facebook, we then discussed the affordances of the first version of the future interface. In connection to the aforementioned example, this step translated to discussing how information gleaned from patients’ Facebook data can be supported by Facebook’s affordances such as tagging, messaging, and *friending*. The frequency of tagging others in a post, the volume of private messages patients exchanged with others, and the number of newly added Facebook friends could be indicative of social interaction in a patient’s life, a critical dimension of most patients’ treatment goals and illness management.

**Table 2:** The first needs-affordances analysis.

Needs (treatment goals)	Affordances	
	Social media (Facebook)	Sketch (prototype)
Reduce the risk for relapse (comply with medication and attend all appointments)	N/A^a^	N/A
Establish social network (increase social interaction and share emotional vulnerability with a new friend)	Social and emotional	Inferring the level of social interactions using Facebook data
Improve existing relationships (apologize for past transgressions and ask for support)	Social and emotional	Inferring the level of social interactions using Facebook data
Reduce/eliminate alcohol and/or drug use (do not buy alcohol, limit consumption to one drink per day, and avoid situations where drugs are present)	Identity	Inferring alcohol-/drug-related behaviors using Facebook data
Get 7 hours of sleep (improve sleep hygiene)	Functional	Inferring sleep hygiene
Eliminate depressed mood (reduce negative cognitions and increase positive activities)	Identity, social, and emotional	Inferring the level of depressed mood
Reduce impairing anxiety (approach feared situations)	Identity, social, and emotional	Inferring the level of anxiety

^a^N/A: not applicable.

**Figure 1:**
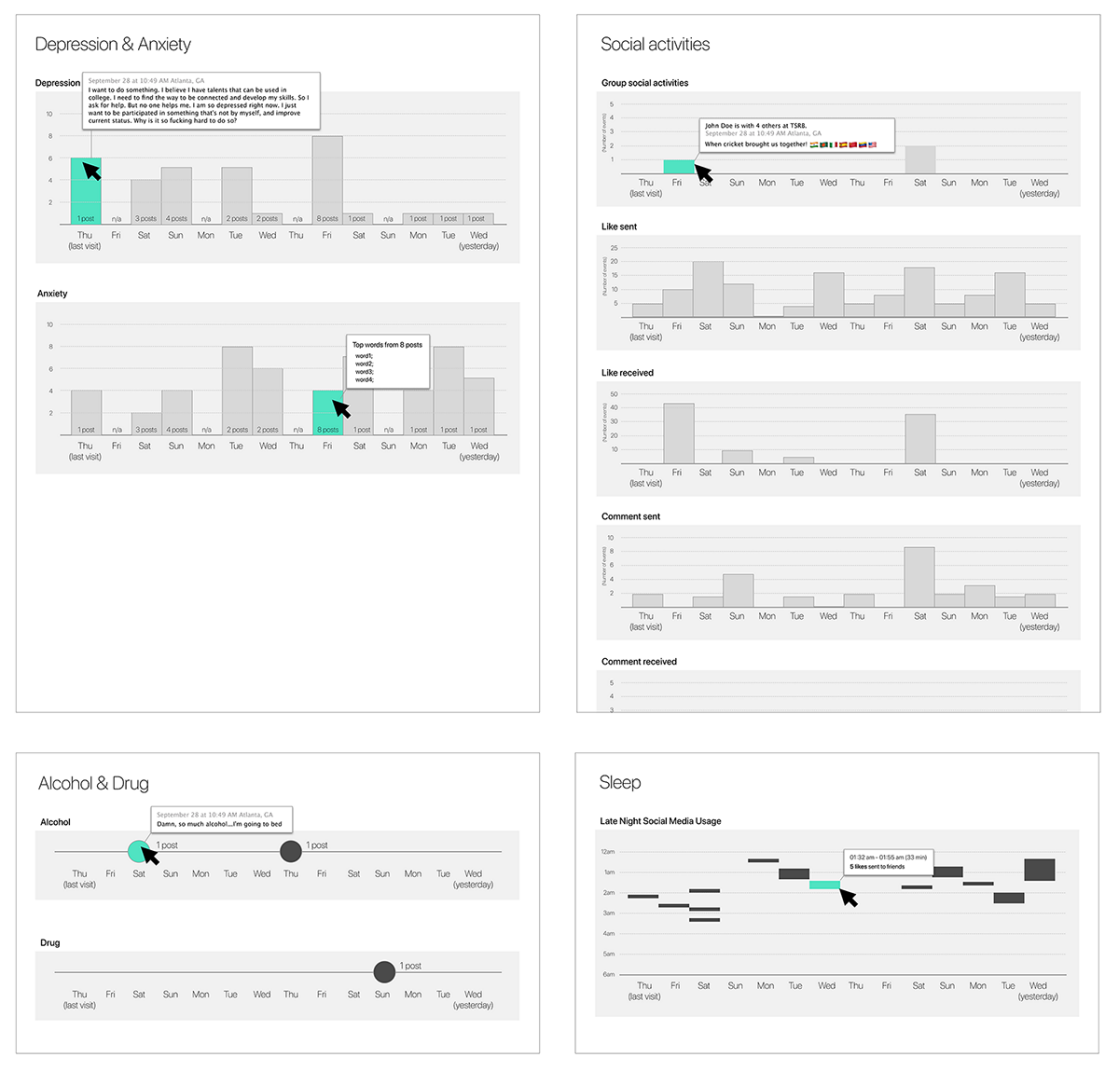
The first prototype: a card-type interface for treatment goal tracking.

In addition to the needs identified earlier, we referred to previous work that visualized patient-generated data for clinicians [41-43]. Previous studies used a dashboard design including a card-type interface where one card represents one type of data/information and users can modify the order and types of cards on the dashboard. We also adopted their design directions because a card-type interface provides flexibility, so researchers can iteratively add and change different types of information. Similar to the previous work, our first sketch (Figure 1) is a card-type visualization, where each card includes the level of the attribute, such as depression, anxiety, social activities, alcohol and drug use, and sleep—all dimensions identified by the clinicians to be important components of tracking common treatment goals in schizophrenia. For depression and anxiety, we envisioned a scale for each symptom that a machine learning approach could automatically infer from the posts (eg, based on the algorithm developed in a study by Saha et al [44]). If a clinician intended to read the actual posts for a specific day, they could hover their mouse over the bar and read the posts on the pop-up layer. For social activities, the page was mainly based on the social affordances of Facebook, such as likes, check-ins, and comments. For the alcohol and drug card, we assumed our interface to identify alcohol- and drug-related posts and present them as a circle on the timeline. For sleep, because social media may not be the best proxy for measuring sleep patterns, we chose to present social media activities after midnight, which may imply some level of sleep hygiene—previous work by De Choudhury et al [13], for instance, found that the degree of night-time social media activity, operationalized as an *insomnia index*, was positively correlated with increased depression risk.

Taken together, clinical collaborators were able to see some value in this form of visualization while pointing out areas of improvement; they pointed out that clinicians may not have time to review all the different cards during an ongoing session with a patient. Furthermore, they noted that they are likely to be most interested in learning about trends around when and how specific mental health attributes showed a change for the patient in question. They also clarified that social media activities related to the quality of life measurement can be interesting to them [15]; hence, a goal of this second prototype was to explore how to present such information derived from the social media data of patients.

### A Clinical State and Trend Visualization Interface

Incorporating feedback from clinical collaborators, we developed the second version of our sketch. This interface is a desktop app that clinicians can access using their personal machines during appointments. The interface consists of 3 different components: the first area is the left menu, which presents information about the patient and clinicians as well as a tree menu of different pages (*Mood*, *Cognition*, *Social Functioning*, and *Language*); the second area is the main content of the page, an overview dashboard as default; and the third area shows long-term trends of all relevant attributes at the bottom.

To arrive at the different components of this interface, in addition to needs-affordances analysis, the widely studied stress-vulnerability model [45] of schizophrenia informed the process of selecting and organizing the additional pages of mood, cognition, social functioning, and language. The model assumes that individuals have an intrinsic level of psychosocial vulnerability, which, in turn, can decide the level of impact realized from different, external, and stressful events. When their vulnerability is high enough and the stressor is strong enough, they will experience transient intermediate states that can be characterized by processing capacity overload, autonomic hyperarousal, and impaired processing of social stimuli. Transient intermediate states can negatively impact a person’s social environment, which can result in repetitive experiences of these states. If this harmful cycle continues until the transient intermediate states surpass an individual’s threshold, it can cause the development of schizophrenic symptoms. Therefore, determining vulnerability is important in assessing and treating people who are suffering from schizophrenia. Previous work has identified various attributes as markers of psychosocial vulnerability: mood, cognition, linguistic style, social functioning, semantic disorganization, syntactic disorientation, and others [46,47], corresponding to each of which we created a page in our second prototype.

In addition, recall that a necessity identified during the discussion of the first prototype centered on clinicians’ desire to learn about patients’ quality of life attributes. Typically, clinicians track such attributes via unstructured conversations with their patients during visits, and it can range over a variety of topics, such as information about their romantic life, work, family, socialization activities, and so on. Inclusion of the *Mood*, *Cognition*, *Social Functioning*, and *Language* pages in this prototype can thus enable tracking such trends over short and long periods in patients’ Facebook data.

We now describe the different components in detail below.

**Figure 2:**
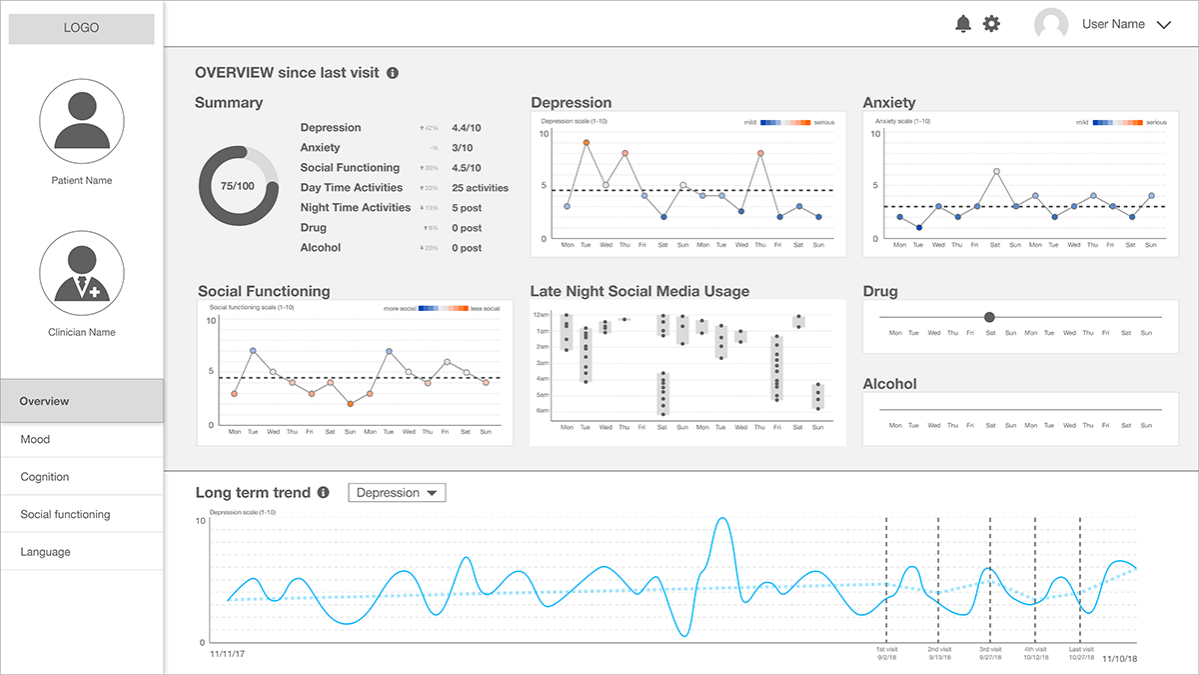
The second prototype: a clinical state and trend visualization interface – "Overview" dashboard.

The overview page, in particular, shows a default view; as shown in Figure 2, the interface uses simple visualization techniques (eg, dot, bar, and line charts) to provide insights that can support common treatment goals, such as learning about trends and monitoring changes related to depression, anxiety, social functioning, drug and alcohol use, and sleep hygiene, since the patient’s last clinical visit.

**Figure 3:**
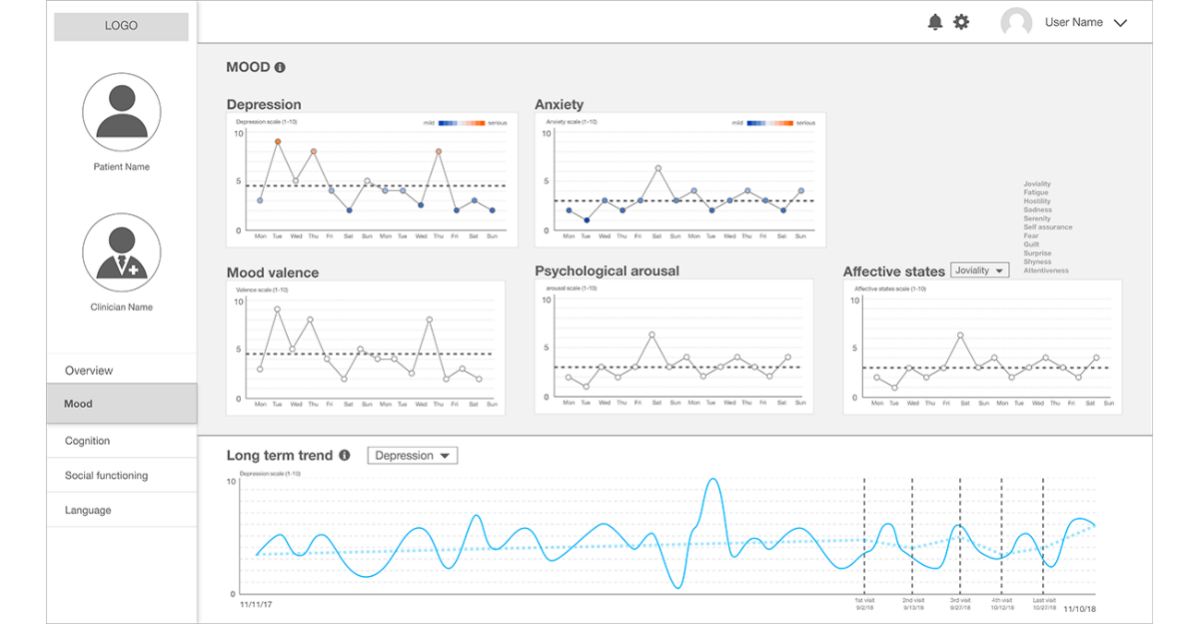
The second prototype: a clinical state and trend visualization interface – "Mood".

Moving on the specific pages in this interface, first, Figure 3 shows the *Mood* category, which provides detailed information about mood and affective states that can augment depression and anxiety information shown in the summary page. Following psychology literature, we provided the level of the 2 different facets of mood—valence (the extent of positivity or negativity of mood) and arousal (the intensity of mood) [48]—and the level of 11 different affective states, such as joviality and fatigue, which have been reliably inferred from computational analyses of social media data in previous work [49]. Owing to the complexity of the nature of mood and affective states, we created simple line graphs for each dimension of those categories.

**Figure 4:**
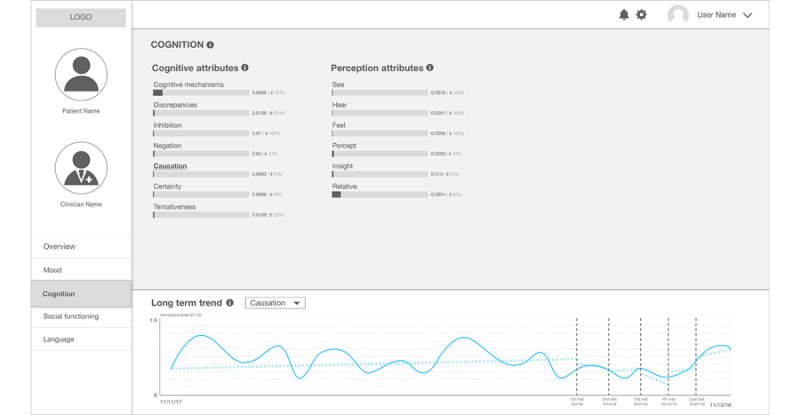
The second prototype: a clinical state and trend visualization interface – "Cognition".

Another category that originated from the stress-vulnerability model, *Cognition*, is important in understanding schizophrenia because the model defines stress as the perceived discrepancies between an individual and the environment surrounding the individual [45]. The additional page (Figure 4) provides scores related to the cognitive processes based on the well-validated computerized text analysis tool, Linguistic Inquiry and Word Count (LIWC) [17,50]. The validity of LIWC was initially approved by the level of agreement between human judges and LIWC’s objective word counting [51], and many studies using social media posts show that LIWC can be applicable to understanding mental health attributes [15,17,52].

**Figure 5:**
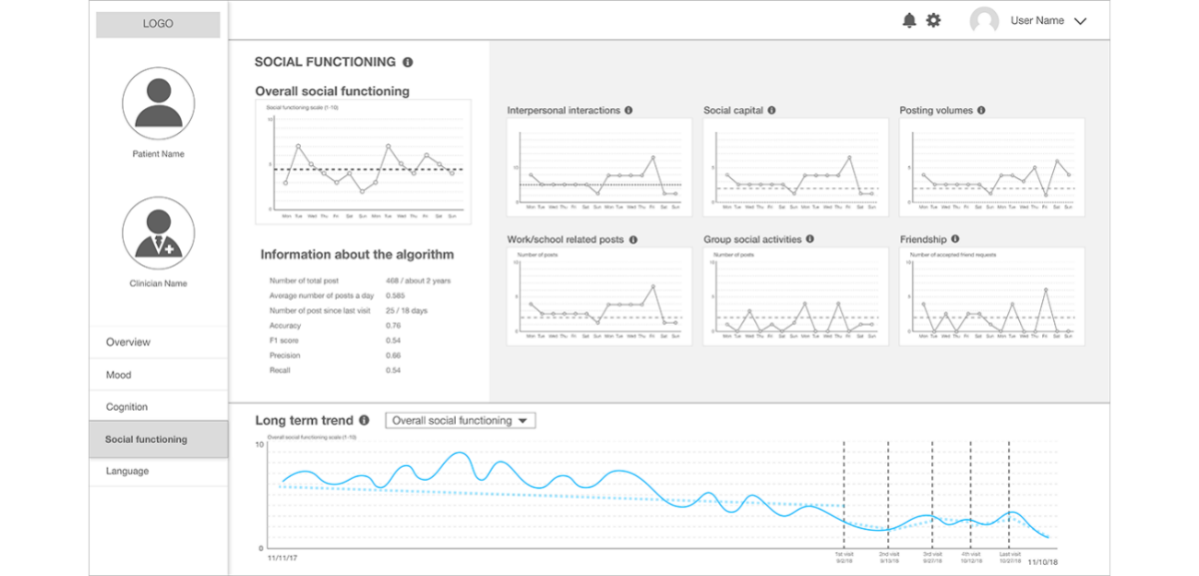
The second prototype: a clinical state and trend visualization interface – "Social Functioning".

Next, *Social Functioning* provides 6 different levels of social media activities that can function as features of a hypothetical machine learning algorithm that can calculate the overall social function score (Figure 5). We selected interpersonal relationships that are accessible on Facebook (number of posts and number of friends) as well as work- and school-related posts because the clinician researchers highlighted the importance of being able to return to their daily activities. Social media activities include the number of posts they have written, the interactions with other Facebook users, work- or school-related posts, and their number of friends.

**Figure 6:**
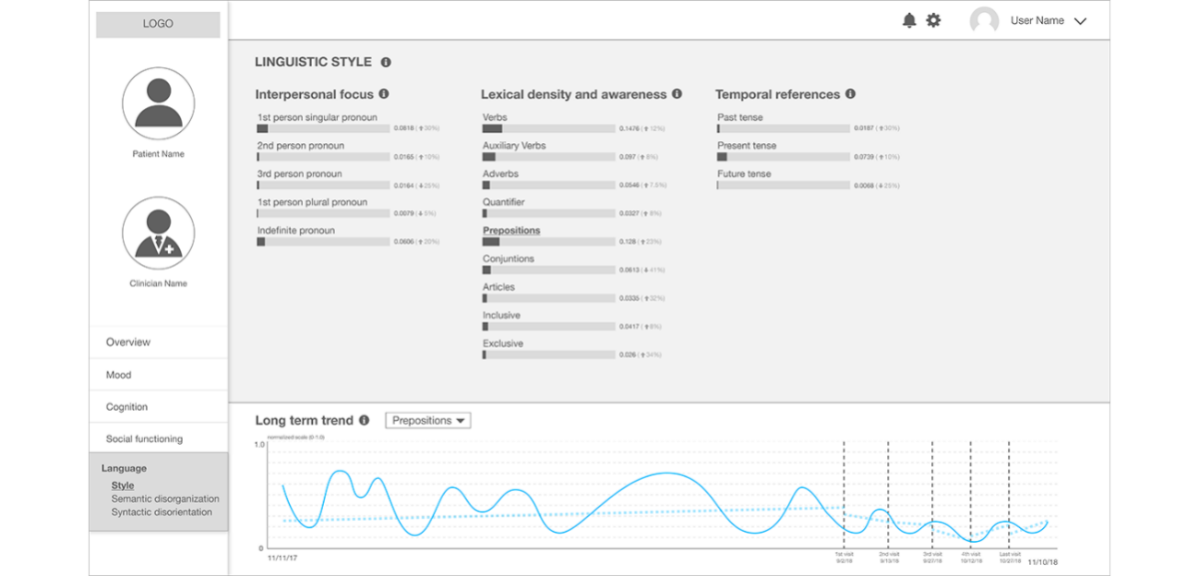
The second prototype: a clinical state and trend visualization interface – "Linguistic Style".

Among the different *Language*-related pages, the linguistic style page (Figure 6) includes some categories from LIWC. The scores for a specific category (eg, 0.08 for first-person singular pronouns) are calculated from the aggregated texts they have posted on Facebook since their last clinical visit; this implies the fraction of words in those texts that are first-person singular pronouns. Previous work has shown that people who self-disclose themselves as patients with schizophrenia show different patterns in pronoun use [17]. The bottom of the page, the long-term trend window, shows temporal changes of a selected category of linguistic style (shown for preposition use) since the patient created their Facebook account.

**Figure 7:**
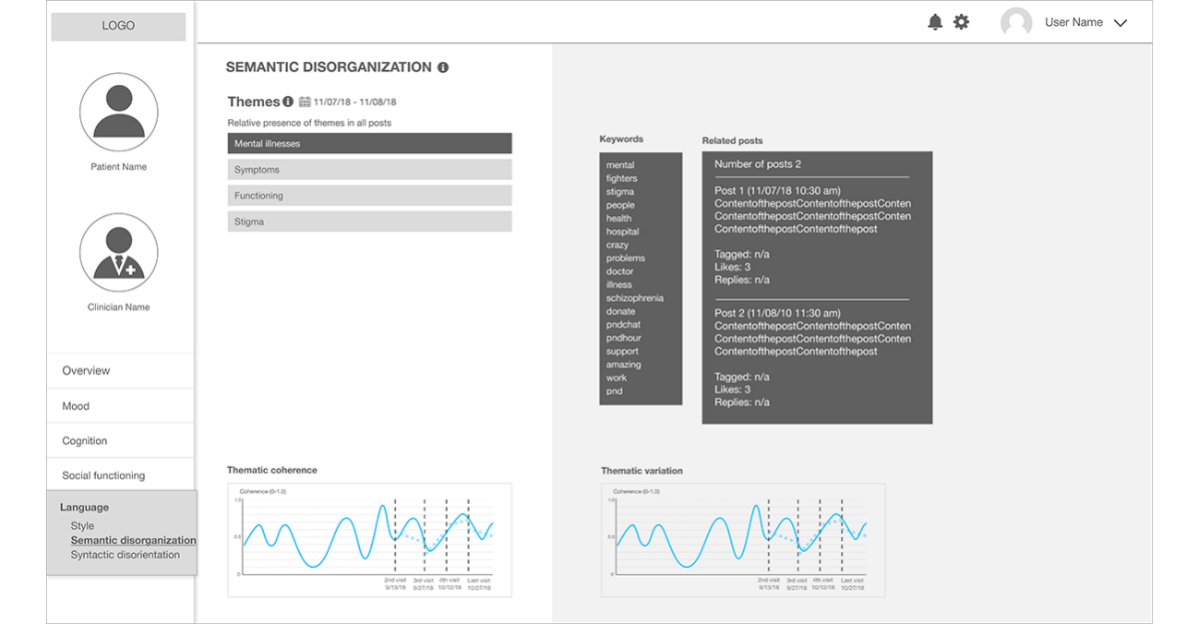
The second sketch: a clinical state and trend visualization interface – "Semantic Disorganization".

Another *Language* page in this prototype, entitled *semantic disorganization* (Figure 7), shows the result of topic modeling applied to patients’ Facebook data. Topic models are algorithms for discovering the latent themes that pervade a large, and otherwise unstructured, collection of documents [53]. We used a visualization technique to use topic models as a navigator that enables users to explore the hidden structure that the algorithm reveals [53]. In Figure 7, the themes identified by the algorithm are listed. As the themes are grouped in a quantitative way, the label of the theme is a group of prevalent words in the theme rather than an intuitive, interpretable title. We envision that human annotation of the label of the theme can provide more meaningful/interpretable information, as has been leveraged in previous work [17]. When users click a theme on the list, the words in the theme and the actual posts in the theme are shown on the right side of the page.

**Figure 8:**
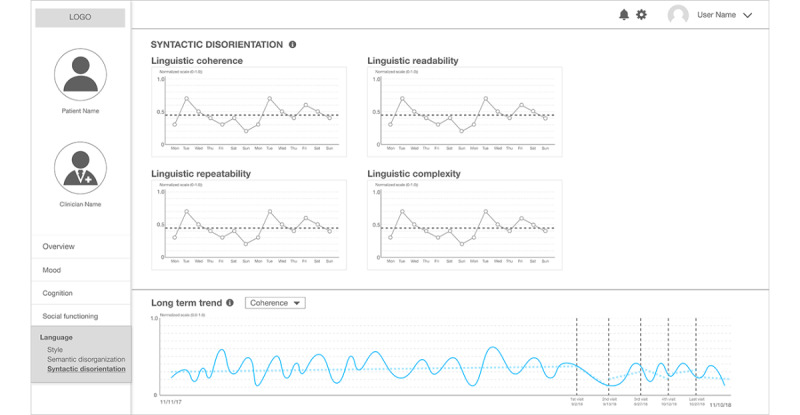
The second prototype: a clinical state and trend visualization interface – "Syntactic Disorientation".

Figure 8 presents the final page within the *Language* category, entitled *syntactic disorientation*, which contains information about linguistic coherence, readability, repeatability, and complexity. We have added this information because of empirical evidence from the literature. Previous work that used natural language analysis models of linguistic change proved that social media users, who self-disclosed themselves as patients with schizophrenia, showed higher repeatability and complexity while, at the same time, lowering coherence and readability when compared with a control group [17]. We selected a simple line graph to maintain the coherence of the visual elements throughout the sketches.

Owing to the elaborate nature of this prototype, clinical researchers provided several rounds of feedback, spanning multiple weeks. Broadly speaking, they appreciated the richness of the information conceptualized to be presented at the interface, which spanned both common treatment goal markers as well as other potentially useful information that can be gleaned from patients’ social media data, which, in turn, could be relevant to gather an understanding of a patient’s quality of life. At the same time, they were skeptical about maintaining the linguistic style page because they felt that our potential users may not have the time to review these highly elaborate different bar charts; may not be familiar with specific language analytic tools such as LIWC, despite being commonplace in the research literature; and may lack knowledge of how the different linguistic analyses were performed. Consequently, they may not be able to interpret the line graphs in a clinically meaningful way. However, clinical researchers have found some value in the linguistic style page; for example, they believed that the temporal reference category, such as past tense, present tense, and future tense, can be meaningful because it is related to understanding the future orientation of patient’s thoughts; future orientation can often indicate an improvement in mental health state [54].

Furthermore, the detailed navigation of patient postings that topic models provide intrigued clinical researchers; the topic model allowed clinical researchers to start thinking about psychosis-related symptoms. Although we did not discuss psychosis-related vulnerability attributes in the first needs-affordances analysis (Table 2), the clinical researchers realized that this information can help lead to a better understanding of their patients. Therefore, we decided to discuss more deeply how we can cover psychosis-related symptoms of schizophrenia in our next iterations of the prototype. We believe this is one of the positive results of the iterative co-design of prototypes; conceptualizing what we need from new technology is difficult even using needs-affordances analysis because domain experts have to ideate several different possible needs while dealing with an unfamiliar and complicated concept for a new, futuristic technology. As a result, the sketches provide a chance to both reflect on potential needs and consider missing needs. In our case, the second prototype focused on mood- and social functioning–related information, but the resulting sketch that highlighted the potential usefulness of the information related to psychosis was missing. Therefore, we decided to conduct a second needs-affordances analysis focusing on the symptoms of schizophrenia patients, which led us to develop a new sketch.

### A Clinical Summary Dashboard

As noted earlier, because we discovered the value of psychosis-related symptoms after reviewing the second version of the sketches, before iterating over and creating the next sketch, we conducted a second needs-affordances analysis (Table 3). During this needs-affordances analysis, the clinical researchers first provided a list of symptoms of interest that are considered expert needs, and then, HCI researchers identified the affordances of platforms and the future interfaces that can correspond with the symptoms. For instance, for symptoms associated with psychosis, the clinical researchers have made a list of symptoms, such as delusions, preoccupations, and hallucinations. We assumed that these symptoms could be inferred from linguistic characteristics and interaction patterns in their Facebook use because of the identity and cognitive affordances of Facebook, as shown in previous computational work on social media and schizophrenia [17].

This second needs-affordances analysis produced 3 groups of expert user needs—symptoms associated with psychosis [46], symptoms associated with mood/anxiety subject to the comorbidities of schizophrenia [55], and social functioning [56]. The first group, symptoms associated with psychosis, was the most deviant part from the previous prototype. The latter 2 groups were similar to treatment goals, such as eliminating depressed mood and improving existing relationships, but as a positive outcome of this iterative process, we were able to make a more detailed list of information needs of mental health clinicians that were represented by the clinical researchers in our team.

From the same needs-affordances analysis, we also learned that clinicians may wish to have an extremely simple summary of social media analysis. Clinical researchers suggested just letting them know whether the severity of symptoms has changed since the last visit using plain text, rather than visuals such as bar graphs. The purpose of the plain text summary is to provide both concise information and opportunities to further explore details when they think the change is interesting, from the perspective of a particular patient meeting their desired treatment goal(s).

**Table 3:** The second needs-affordances analysis.

Needs		Affordances	
		Social media	Future interface
**Symptoms associated with psychosis**			
	Delusions	Identity and cognitive	Measuring the severity of delusion
	Preoccupations	Identity and cognitive	Measuring the severity of preoccupations
	Hallucinations	Identity and cognitive	Measuring the severity of hallucinations
	Paranoia	Identity and cognitive	Measuring the severity of paranoia
	Thought disorganization	Identity and cognitive	Measuring the level of thought disorganization
	Shifting or fast thoughts	Identity and cognitive	Detecting extreme/abnormal thought shifting
	Health concerns	Identity and cognitive	Detecting extreme/abnormal health concerns
	Awareness	Identity and cognitive	Inferring levels of awareness of their illnesses/symptoms
	Changes in concentration	Identity and cognitive	Detecting extreme/abnormal changes in concentration
**Symptoms associated with mood/anxiety**			
	Anxiety/worries	Emotional	Measuring levels of anxiety
	Low mood/high mood/mood swings	Emotional	Inferring mood
	Feelings of guilt/shame	Emotional	Measuring affective states (guilty)
	Suicidal/homicidal thoughts	Emotional	Detecting suicidal thoughts
	Grandiosity	Emotional	Detecting exaggerated self-confidence
	Irritability/hostility/aggression	Emotional	Measuring affective states (hostility)
	Decreased energy/increased energy	Emotional	Measuring affective states (fatigue and attentiveness)
	Sleep changes	Emotional	N/A^a^
	Feelings about the future	Emotional	N/A
	Hopelessness/hopeful/worthlessness	Emotional	N/A
	Appetite changes	Emotional	N/A
**Social functioning**			
	Engaging in/initiating pleasurable activities	Emotional and social	Counting the number of “interested in” events
	Engaging in social activities/avoiding people/socially isolated	Emotional and social	Counting the number of “check in” posts
	Motivation for school/work/volunteering	Emotional and social	N/A
	Strength of social ties	Emotional and social	Number of interactions (messages, likes, taggings, reply, and posts)
	Role function (going to work/school/volunteering)	Emotional and social	Detecting posts related to work/school
	Sexual interest/dating	Emotional and social	Counting words related to sexual interest

^a^N/A: not applicable.

**Figure 9:**
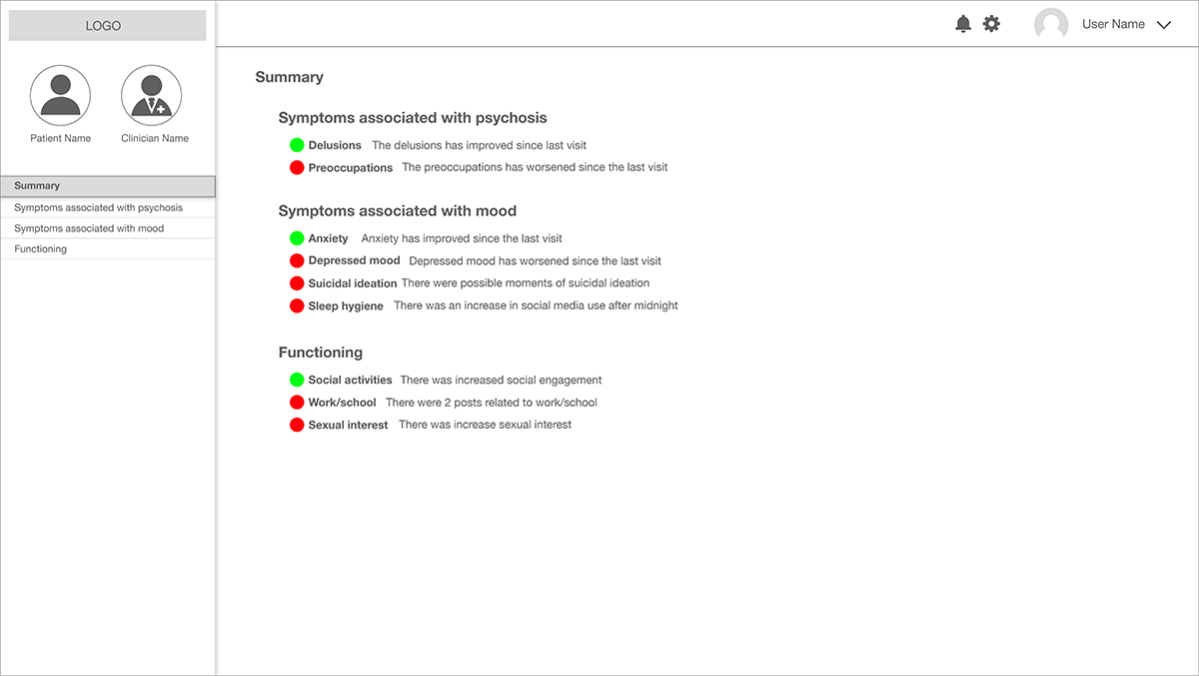
The final (third) version of our prototype: A clinical summary dashboard.

Figure 9 shows the first page of this summative dashboard. Symptoms from the second needs-affordance analysis that have changed since the last visit are shown using colored circles and plain text. The green circles indicate the symptoms that have improved since the last visit, and the red circles indicate the deterioration of a symptom. The plain text tells which symptoms have changed, and if applicable, what the changes were (eg, there were 2 posts related to work/school for the work/school category in the illustration of Figure 9). We assumed that symptoms that have changed since the last few visits can be interesting starting points for clinicians; if they want to look at the details, they can click and see bar charts that are similar to those in Figure 2.

## Discussion

### Lessons Learned

The iterative nature of our co-design process enabled us to expand the potential use of the prototype. Clinical researchers’ immediate responses to the first sketches were ambivalent: they were positive about being able to gather more fine-grained collateral information about their patients, which may help them overcome the weaknesses of current self-report–based information gathering processes. At the same time, they were concerned about whether the potential information could overload their work practices, as social media–derived information could be seen as another layer of noise. After they reviewed the revised sketches that were more well aligned with their needs and concise enough to be used in a limited amount of time, they started to consider other details. For example, they were highly interested in social functioning–related information in general.

Clinical researchers also envisioned potential alternative uses of the tool that can support their work practices. In earlier meetings, the clinical researchers suggested that future tools could be more beneficial for therapists than psychiatrists because therapists usually have longer interactions with patients that can be aided by the proposed technology. However, after they saw the second prototype—the clinical state and trend visualization interface (Figure 2)—they were of the opinion that they may be able to use the dashboard to facilitate conversations between the treatment team—a combination of psychiatrists, psychologists, therapists, and social workers—which meets periodically in smaller, more cutting-edge, care-centric mental health clinics.

In addition, our co-design activities essentially revealed an *information abstraction gap*, that is, a gap between the *computational abstraction of patients’ Facebook data* and the *clinical abstraction of patients’ states*. We introduced computational approaches within our designs because clinicians expressed the busy nature of their job, and patient social media data, although perceived to be valuable, can be gigantic. Computational approaches allow converting raw social media data into a more consumable unit of information (ie, from *the total number of posts* to *the number of depression-indicative posts*). These types of abstracted information derived from computational approaches were successful when the information was intuitive and compatible with clinical concepts. One of the most successful cases was the measures in the overview page in the second prototype (Figure 2). However, at the same time, aside from this potential, clinical researchers were skeptical in other measures that are based on either machine learning algorithms (eg, the performance metrics of the algorithms) or psycholinguistic analysis (eg, LIWC scores) because interpreting these measures is not a part of the current clinical culture; some measures may not be clinically meaningful or align with how a clinician assesses a patient’s mental health state, whereas others may not support their current workflows.

Therefore, our co-design focused on bridging the gaps between *what the computational approaches can provide* and *what clinicians can actually use at the point of care*. The 2 needs-affordances analyses crystalized the latter part—how they abstract the diverse types of patient information into clinical categories. The second analysis yielded the 3 categories that summarized clinicians’ mental models of how they navigate patients’ information to make a clinical decision. The second and third prototypes explored how computational approaches can be matched to the clinicians’ mental models. The simplified visual representation in the third prototype reconciled the vast amount of data with the clinicians’ needs for immediate, actionable insights from new technology.

Although we were able to result in a meaningful bridge between the gaps, there are still interesting aspects to explore; most importantly, the flow of patient-clinician consultations varied based on the types of clinicians, patients, and treatment strategies. In addition, information abstraction gaps can exist between stakeholders, such as clinicians, patients, the managers of hospitals, family members, and other caregivers. We need to explore, in future work, how computational abstraction can support/hamper communications between the stakeholders. Finally, the information abstraction gap is a sociotechnical phenomenon that is situated in cultures, policies, and laws surrounding the computational artifact and the users of the artifact. Therefore, future research should take into account institutional and cultural support to make new technologies implementable and sustainable.

### Comparison With Previous Work

The concept of the use of mental health insights from social media is relatively novel. Fisher et al [57] provided case reports where clinicians had begun to incorporate patients’ electronic communications, such as social media in their care. For instance, these authors reported that one clinical team reviewed an outpatient’s Facebook feed because the patient was vulnerable to self-harm and violent behavior. In the face of these isolated incidents, to understand systematically how social media data may augment treatment for mental health conditions, Hobbs et al [58] surveyed 115 outpatient psychotherapy clinicians associated with McLean Hospital in Belmont, Massachusetts. They found that 61.7% (71/115) of clinicians reported viewing at least one patient’s social or electronic media as part of psychotherapy, and 92% (65/71) of those endorsed being able to provide more effective treatment using this information.

However, despite revealing these potentials, design opportunities for social media at the point of care have not yet been explored. Our work is based on evidence in recent literature that explored the use of digital phenotyping data [59-61]. For example, Luo et al [60] identified dietitians’ needs for patient information regarding food, reflection, symptoms, activity, and physical state. Similar to this work and others, our co-design approach also identified clinicians’ needs for more objective patient information and contextualized the use of social media for this purpose. Our findings regarding clinicians’ needs for symptoms and social functioning in patients with schizophrenia, in fact, resonate with similar observations in previous research. Specifically, we suggest that patient information can be grouped as symptoms related to psychosis, symptoms related to mood/anxiety, and social functioning. Furthermore, to address the cognitive overload of clinicians that has been identified in previous work [62], our co-design approach resulted in a clinical summary view that provides changes in an extremely simple form.

### Limitations and Future Research

Even though we were able to learn valuable lessons from the co-design approach, we acknowledge that there are certain limitations in this work. First, we note that the computational approaches that are considered in our prototype are in the early stages of research and require more rigorous and clinical validation before being deployed in clinical settings. Second, the user needs we considered are the potential user needs that are inferred by a small number of clinical researchers. It is possible that their projections of user needs may not fully represent our target groups’ opinions. We believe this limitation can be overcome with subsequent studies to gather user needs with a representative group of participants. It should be noted that we have not evaluated our prototype with potential users as a form of user study that can yield diverse design implications and guidelines. As mentioned earlier, this is the very first stage of a long-term project where we envisioned a future tool as an interdisciplinary working group, and we considered using a long-term co-design activity for the first step. We believe this first step has informed our team to proceed to the next stage of our research, including building a medium-fidelity prototype and a user study using the functioning prototype.

In addition to the abovementioned points, we also acknowledge that patient perspectives and ethical considerations are massive issues surrounding the concept of using patient-generated data, such as with our proposed technology. That said, as a formative step, we wanted to ensure that clinicians perceived the technology in question to have clinical value before involving patients directly. We know that patients’ autonomy and agency are critical in designing a technology that uses sensitive information from vulnerable populations. Future endeavors will augment these insights by including patients in an iterative design process to explore their perspectives, especially their autonomy and agency.

As a next step, we intend to conduct a qualitative evaluation study using our prototype, where we will introduce the concept of the future interface to a diverse set of clinicians. At the same time, we will seek their opinions about adopting this future technology, potential barriers to using this concept, and other concerns. We believe our prototypes can be useful for exploring these opinions because we co-designed these prototypes with domain experts who can represent our potential user group. This was the result of a relatively long-term discussion between HCI researchers and domain experts, which enabled them to educate each other on their own expertise, ideate future directions, and discuss remaining concerns. It might not be possible to have this deep discussion during traditional user study sessions, such as one-time, one-hour long interviews. We believe our future participants in the user study sessions using our prototypes can provide rich responses to questions regarding the adoption of this concept because our prototypes can be well aligned with their potential needs and work practices. Specifically, we are interested in questions related to usability (whether they can understand and use the prototype without problems), compatibility (whether the tools can be used in their work practices without frictions), and perceived clinical usefulness (whether they foresee the clinical value of using the prototype). We hope this co-design process and future qualitative study using the prototypes can lead us to develop a usable and useful clinician-facing tool in the future that can be used efficaciously at the point of care.

## Abbreviations

HCI: human-computer interaction
LIWC: Linguistic Inquiry and Word Count
lo-fi: low-fidelity
